# Water Filtration in America

**Published:** 1901-01-12

**Authors:** 


### WATER FILTRATION IN AMERICA.

The efficacy of filtration on the large scale as a means of purifying water has received a good illustration in the apparent effect upon the death rate which has followed the installation of a filtration plant at Albany, in the United States. According to a report in the New York *Medical Record*, the greater portion of the water-supply of Albany has of late years been drawn from the river Hudson, which was seriously contaminated by local sewage as well as by that coming from Troy, Cohoes, and other cities, and the typhoid rate has been excessive. The filtering plant erected supplies an average of 12,000,000 gallons per day. The effect of the water-supply on the health of the city has been quick and decided. The average number of deaths from typhoid fever in Albany for nine years up to 1899 was 85 in a year, and from January to August 1899, while the city was still using the unfiltered water, there were 71 deaths from this disease. From August 1st, however, when the filtered supply began, to July 1st, 1900, that is for nearly a year, there were only 24 deaths from typhoid, against 85, the average for preceding years. The Albany people are very proud of their filtration plant, and quite properly so.

